# Electron-Rich Triazine-Conjugated Microporous Polymers for the Removal of Dyes from Wastewater

**DOI:** 10.3390/molecules28124785

**Published:** 2023-06-15

**Authors:** Bao-Ning Li, Xing-Long Zhang, Xiao-Hui Bai, Zhen-Jie Liang, Jian Li, Xiao-Yong Fan

**Affiliations:** 1 School of Chemistry and Chemical Engineering, Yulin University, Yulin 719000, Chinabxh0727@163.com (X.-H.B.); lijian5220@163.com (J.L.); fanxy@yulinu.edu.cn (X.-Y.F.); 2 School of Chemistry, Sun Yat-Sen University, Guangzhou 510006, China

**Keywords:** conjugated microporous polymers, adsorption, dyes, wastewater

## Abstract

Conjugated microporous polymers (CMP) as porous functional materials have received considerable attention due to their unique structures and fascinating properties for the adsorption and degradation of dyes. Herein, a triazine-conjugated microporous polymer material with rich N-donors at the skeleton itself was successfully synthesized via the Sonogashira–Hagihara coupling by a one–pot reaction. These two polymers had Brunauer–Emmett–Teller (BET) surface areas of 322 and 435 m^2^g^−1^ for triazine-conjugated microporous polymers (T-CMP) and T-CMP-Me, respectively. Due to the porous effects and the rich N-donor at the framework, it displayed a higher removal efficiency and adsorption performance compared to cationic-type dyes and selectivity properties for (methylene blue) MB^+^ from a mixture solution of cationic-type dyes. Furthermore, the T-CMP-Me could quickly and drastically separate MB^+^ and (methyl orange) MO^−^ from the mixed solution within a short time. Their intriguing absorption behaviors are supported by ^13^C NMR, UV−vis absorption spectroscopy, scanning electron microscopy, and X-ray powder diffraction studies. This work will not only improve the development of porous material varieties, but also demonstrate the adsorption or selectivity of porous materials for dyes from wastewater.

## 1. Introduction

Recently, porous organic materials have become a hot topic due to their numerous advantages, such as diversified structure, huge surface, tunable porosity, et al. [1,2,3,4,5]. Over the past few years, metal–organic frameworks (MOFs) [6], covalent organic frameworks (COFs) [7], and porous organic polymers (POPs) [8] have developed rapidly. MOFs and COFs have robust and ordered structures but low physicochemical stability, while conjugated microporous polymers (CMP) have high stability and an easy preparation method [9]. Thus, designing a functional porous material that can combine the advantages of different porous materials, called triazine-conjugated microporous polymers (T-CMP), may be more meaningful for the development of porous organic materials [10,11,12,13].

Nowadays, water pollution has directly affected human health [14,15], owing to numerous dyes released by factories, and the different types of dyes have become different sources of pollution; therefore, effective methods are needed to deal with the toxic chemical dyes from the wastewater [2]. Different methods, such as precipitation, ultra-filtration, ion exchange, coagulation, and electro-catalytic degradation, have been adopted for the adsorption and degradation of these organic pollutants [16,17,18,19,20]. Apart from these methods, adsorption, photocatalytic degradation, and chemical degradation are considered the most economical and efficient ones to control water pollution from dyes [21].

Conjugated microporous polymers are considered the best candidates employed in various environmental and energy-related systems for their stable chemical/physical structure, pore walls, pore volume, pore surface area, pore skeleton, and pore technology [12,13]. In past years, different terminologies were used [20], but since an important class of porous materials, CMP–1, was obtained by the Sonogashira reaction between 1,3,5-triethynylbenzene and 1,4-diiodobenzene in 2007 [22], the word “CMP” has become the most popular and well known. CMPs are synthesized under kinetic and/or thermodynamic control with rigid *p*-conjugate structures and three-dimensional networks [23,24]. Additionally, ionic functionalized CMP porous materials also exhibit unique properties, such as high porosity, broad and interconnected pores, and the ability to quickly absorb and desorb ionic dyes, making them more ideal [12,25]. Therefore, the preparation and application of ion-functionalized CMPs have attracted more and more research interest [26,27]. Along with these effects, a series of porous polyionic liquids have been successfully synthesized by template or no template methods [25], revealing the high ion density, high polarization, and strong electrostatic field, which have been widely used in catalysis, adsorption, separation, sensors, actuators, and other fields [14,28,29]. In the past few decades, people have developed various preparation strategies to construct new ionic types of CMPs [30,31]. One of the traditional methods is the cross-coupling reaction between the conjugated building unit and ionic functional monomers [32]. Even if it is an effective method to obtain ionic CMP, it is difficult to make organic salts participate in cross-coupling reactions. Compared with previous methods, the post-synthetic modification (PSM) method has the advantages of high yield, simple monomers, simple operation, and good selectivity, which plays an important role in the construction of the ionic CMP material [33]. The ionic porous nanostructured polymer adsorbents have quick adsorption and ion exchange processes, resulting in high capacity and analyte selectivity for ionic dyes [17,34]. In the process of material preparation, designing specific building blocks with similar functions to construct CMPs has received increasing attention [16,21,35].

Herein, we report two functional triazine-conjugated microporous polymers, which were synthesized by the Sonogashira–Hagihara coupling reaction. The functional polymer materials show excellent selectivity and adsorption for various types of dyes.

## 2. Results and Discussion

### 2.1. Synthesis of T-CMP and T-CMP-Me

**Scheme 1:**
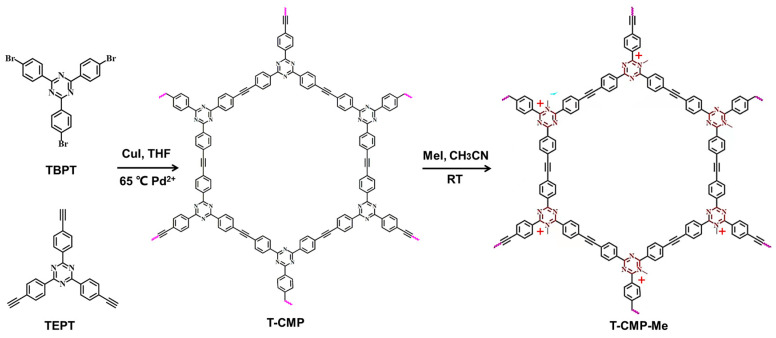
Synthetic strategy for T-CMP and T-CMP-Me.

As shown in Scheme 1, the T-CMP network is synthesized by polycondensation via a Sonogashira–Hagihara coupling reaction [36]. In a typical procedure, the molecules for 2,4,6-tri(p-bromophenyl)-1,3,5-triazine (TBPT) and 2,4,6-tris(4-ethynylphenyl)-1,3,5-triazine (TEPT) are synthesized and characterized (Figures S1 and S2). The general procedure for the synthesis of T-CMP followed the methods in the literature [36]. An ice-cooled mixture solution of 20 mL THF and 20 mL diisopropylamine was added to (0.05 mmol, 27.0 mg) TBPT, (0.05 mmol, 19.0 mg) TEPT, (15.0 mg, 0.08 mmol) CuI, and (84.0 mg, 0.12 mmol) Pd(PPh_3_)_2_Cl_2_, and stirred at room temperature for 20 min. After this procedure, the mixture was stirred at 65 °C for another 48 h. Then, the hot dark-yellow solution was filtered and washed with CHCl_3_, CH_3_OH, and H_2_O to remove the unreacted reactants as well as catalysts, and then the polymer was further purified by Soxhlet extraction with acetone for three days. There was a yield of 98% for T-CMP. In addition, the T-CMP-Me was synthesized by refluxing a mixture of T-CMP and excess MeI in CH_3_CN (40 mL) under an N_2_ atmosphere, according to the literature. Finally, the brown-yellow solid powder was obtained and then dried in vacuo. Additionally, T-CMP-Me was prepared through protonation modification by iodomethane under conventional refluxing conditions [37].

### 2.2. Characterization of T-CMP and T-CMP-Me

**Figure 1:**
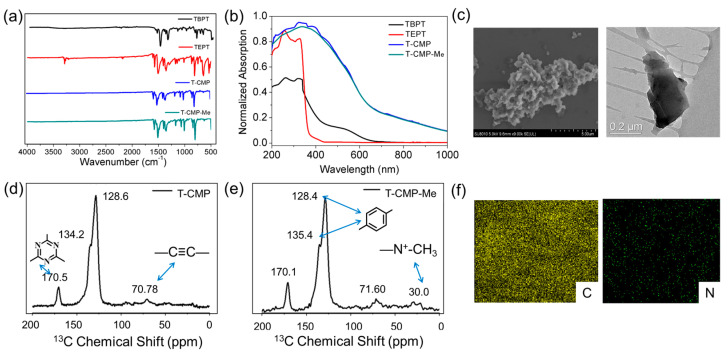
The (**a**) FT-IR and (**b**) solid-state UV/vis diffuse reflectance spectra of T-CMP, T-CMP-Me, and the corresponding monomers. (**c**,**f**) STEM-HAADF and elemental-mapping images of T-CMP. (**d**,**e**) The ^13^C CP/MAS NMR spectra of T-CMP and T-CMP-Me.

The as-prepared polymers T-CMP and T-CMP-Me are further well characterized by FT-IR, ^13^C-NMR, UV/vis, SEM, and TEM, respectively, as shown in Figure 1, Figures S3–S5, and Table S1. The successful connection between TBPT and TEPT is confirmed by the absence of the C-H stretching band of terminal alkyne at ca. 3288 cm^−1^ and the C-Br vibration band at ca. 1060 cm^−1^ in the IR spectrum. In addition, the peaks at 670–751, 1069–1421, and 1583–1596 cm^−1^ are assigned to the wagging and torsion vibrations of benzene triazine stretching vibrations and the −C=N group stretching vibrations of benzene triazine [38], while benzene ring typical absorptions are also observed at 1341 and 1597 cm^−1^, as shown in Figure 1a. The solid UV-visible reflectance spectra show a broader and stronger absorption with a larger reflection edge of 1000 nm compared to the molecules TEPT and TBPT, as shown in Figure 1b, as well as displays the highly conjugated structure of the triazine polymers. From the solid ^13^C-NMR spectra (Figure 1d,e), the alkyne groups, as the important connecting group of the networks, have two obvious peaks at δ = 70.78 ppm; meanwhile, the response at δ = 128.6 ppm could be assigned to the aromatic carbon atoms of benzene. In comparison, the additional peaks at δ = 170.5 ppm are the characteristic signal of benzene triazine rings. Importantly, the resonances at δ = 30.0 ppm belong to the functional methyl group; all these results indicate the successful formation of the network polymers.

The morphologies of the polymer materials T-CMP and T-CMP-Me are further characterized by scanning electron microscopy (SEM) and transmission electron microscope (TEM) (Figure 1c,f and Figure S5). It reveals that T-CMP and T-CMP-Me showed an amorphous ellipsoidal-like in morphology features, respectively. In addition, inductively coupled plasma atomic emission spectroscopy analysis of T-CMP revealed that the C and N contents are 14.29 wt% and 4.21 wt%, respectively, which are close to the theoretical values, as shown in Figure S6 and Table S1. The powder X-ray diffraction (PXRD) patterns give no definite evident peaks (Figure S7), revealing their amorphous nature [28]. Among them, the X-ray powder diffraction pattern of T-CMP shows obvious diffraction peaks at around 7.75° and 20.86°, and the diffraction peaks appearing around 20.86° are attributed to the tendency of interlayer accumulation, according to the previously reported conjugated microporous polymers [1,7,12]. It estimates that the layer spacing of T-CMP is about 4.3 Å through the Bragg equation; at the same time, the X-ray powder diffraction pattern of T-CMP-Me only shows a wide diffraction peak of nearly 22°, which indicates that the interlayer accumulation is affected and the degree of crystallization decreased after methylation. Therefore, we speculate that after methylation, the charge effect is enhanced, which leads to strong repulsion between each of the layers. 

## 3. Porosity Measurements and Gas Adsorption Studies

**Figure 2:**
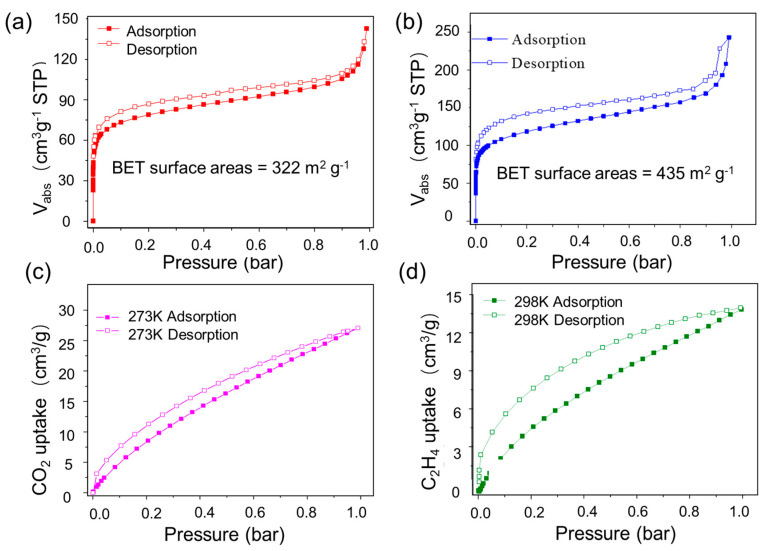
(**a**,**b**) N_2_ adsorption–desorption isotherms (77 K) of T-CMP and T-CMP-Me, respectively. (**c**) CO_2_ adsorption–desorption isotherms (273 K) of T-CMP. (**d**) C_2_H_4_ adsorption–desorption isotherms (298 K) of T-CMP.

The porous structures of T-CMP and T-CMP-Me are further confirmed by nitrogen sorption isotherm measurements at 77 K, which are shown in Figure 2a,b, respectively. The solid materials exhibited typical type I isotherm curves, confirming its microporous framework. Moreover, the TG analysis of the polymers revealed good thermal stability at about 400 °C (Figure S8). The Brunauer–Emmett–Teller (BET) surface areas were 322 and 435 m^2^g^−1^ for T-CMP and T-CMP-Me, with average pore widths of 1.55 and 1.50 nm (Figures S9 and S10), respectively. T-CMP-Me has a larger surface area than T-CMP, which is attributed to the steric hindrance of methyl groups or lamellar charge repulsion [39]. Although the surface area of T-CMP is not large enough, the triazine-conjugated microporous polymers contain a large number of bare nitrogen atoms, which may enhance the adsorption of CO_2_ and some other gases due to the excess electrons. First, the H_2_ absorption at 77 K (Figure S11) was measured, which shows an uptake of 46.88 cm^3^g^−1^. Furthermore, the CO_2_ uptake absorptions at different temperatures were measured, which show uptakes of 27.12 cm^3^g^−1^ at 273 K and 0.39 cm^3^g^−1^ at 298 K (Figure 2c). This revealed that the conjugated polymer would be a candidate for the capture of CO_2_, accounting for the triazine pyrimidine rings, which have good bond energy to CO_2._ Finally, as shown in Figure 2d, the T-CMP porous materials could also be a sorbent for C_2_H_4_; however, it displays a lower absorption for the olifant gases. All in all, the favorable capacity of the various gases was due to the abundant basic pyrimidine rings or the conjugated microporosity of the polymer skeleton.

## 4. Dye Adsorption Performance

**Figure 3:**
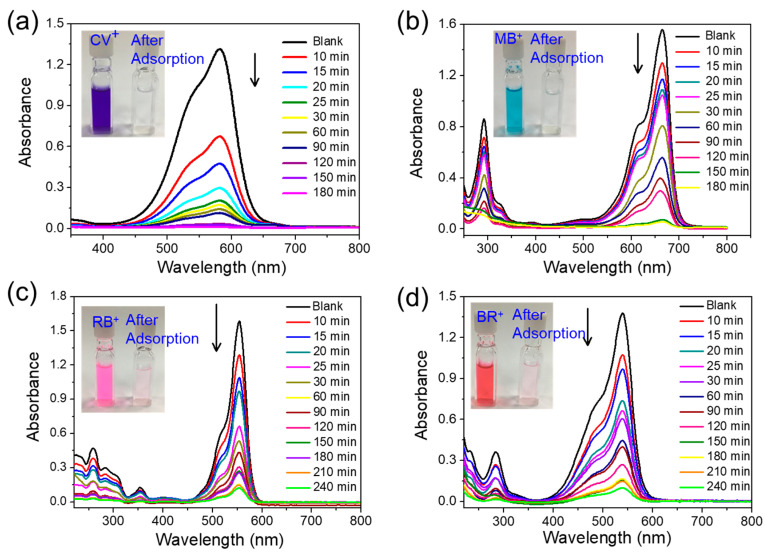
Time-dependent adsorption study of 20 mg/L^−1^ for each kind of cationic dye in 20 mL aqueous solution: (**a**) CV^+^, (**b**) MB^+^, (**c**) RB^+^, and (**d**) BR^+^ for T-CMP (3 mg). Inset photographs show the color change before and after dye adsorption.

The T-CMP and T-CMP-Me have a large number of N-donor sites and pore space inside, which lead to the ability to adsorb dye pollutants from water well. Herein, the material was considered a candidate for the capture of cation dyes from the colorful water solution (Figure 3 and Figures S12–S19). Thus, seven kinds of dyes, namely, cationic-type crystal violet (CV^+^), methylene blue (MB^+^), rhodamine B (RB^+^) and basic red (BR^+^), anionic-type methyl orange (MO^−^) and acid orange (AO^−^), and neutral-type dimethyl yellow (DY) were chosen as the target adsorbent dyes (Table S2). In order to further verify the hydrophilic and hydrophobic properties (Figure S19), the contact angle tests were measured. It can be observed that T-CMP has a better hydrophobic ability, which is consistent with the absorption of dyes. To check the adsorption abilities of T-CMP and T-CMP-Me, T-CMP and T-CMP (3 mg) were added to 20 mL of dye (CV^+^, MB^+^, RB^+^, and BR^+^) solution in water (20 mg/L^−1^). Obviously, the color and the concentration of CV^+^, MB^+^, RB^+^, and BR^+^ changed at different rates (Figure 3). As shown in Figure S17, the near removal efficiency of the cation dyes are in the order of BR^+^ < RB^+^ < MB^+^ < CV^+^, which may be caused by the molecular size or the interaction between the molecules and the T-CMP [40]; the above phenomenon demonstrated that T-CMP would only adsorb cationic-type dyes. Furthermore, for the monoanionic dye MO^−^ or AO^−^, almost no dye was adsorbed by the T-CMP material for a long time (Figures S18 and S19). However, the cationic T-CMP-Me shows obvious adsorbed properties (Figures S15 and S16) due to the electric charge and physisorption effect. For neutral dyes, DY (Figure S19) takes two times as long as the cationic types, owing to their porous effect. 

The above results of UV/vis absorption spectra for different dyes show that the T-CMP and T-CMP-Me materials like to adsorb various types of dyes because the skeleton of the material itself contains N-donors. This is due to the fact that the polymers are rich in electrons, which are conducive to adsorbing the molecular dyes with electron deficiency. Meanwhile, the triazine-conjugated microporous polymers also have natural adsorption properties, which is consistent with the conclusion of gas adsorption.

**Figure 4:**
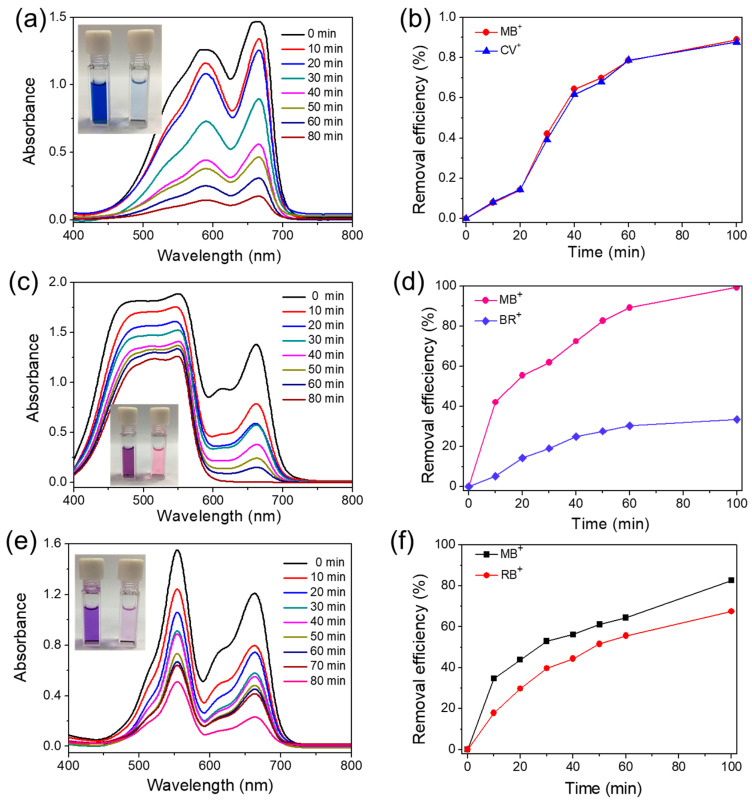
Time-dependent adsorption study of 20 mg/L^−1^ for each kind of cationic dye in 20 mL aqueous solution: (**a**) MB^+^/CV^+^, (**b**) MB^+^/BR^+^, and (**c**) MB^+^/RB^+^ for T-CMP (5 mg). The removal efficiency of each aqueous mixture: (**d**) MB^+^/CV^+^, (**e**) MB^+^/BR^+^, and (**f**) MB^+^/RB^+^. Inset photographs show the color change before and after dye adsorption (removal efficiency = (C_0_ − C_t_)/C_t_; C_0_: the original concentration; C_t_: the concentration at the moment t).

For further study of the phenomenon, we also studied the aqueous solutions containing mixed dyes of MB^+^/CV^+^, MB^+^/BR^+^, MB^+^/RB^+^, MB^+^/AO^−^, and MB^+^/DY, as shown in Figure 4. Owing to the rich N-donor porous framework of T-CMP, obviously, the homologous cationic dye colors of the MB^+^/CV^+^, MB^+^/BR^+^, and MB^+^/CV^+^ mixtures changed from deep blue to light blue, from modena to slightly pink, and from bluish violet to lavender, respectively, in Figure 4a–c. Checking the adsorption selectivity, it was found that only the mixture of MB^+^/BR^+^ changed significantly; the highest absorption peaks at 655 nm of MB^+^ drops quickly compared to the BR^+^; however, the removal efficiency for the mixture of MB^+^/CV^+^ and MB^+^/CV^+^ is changed homogenously. From the above conclusions, the selective cationic dye adsorption suggests the T-CMP could adsorb homologous dyes in a mixture of water pollution.

**Figure 5:**
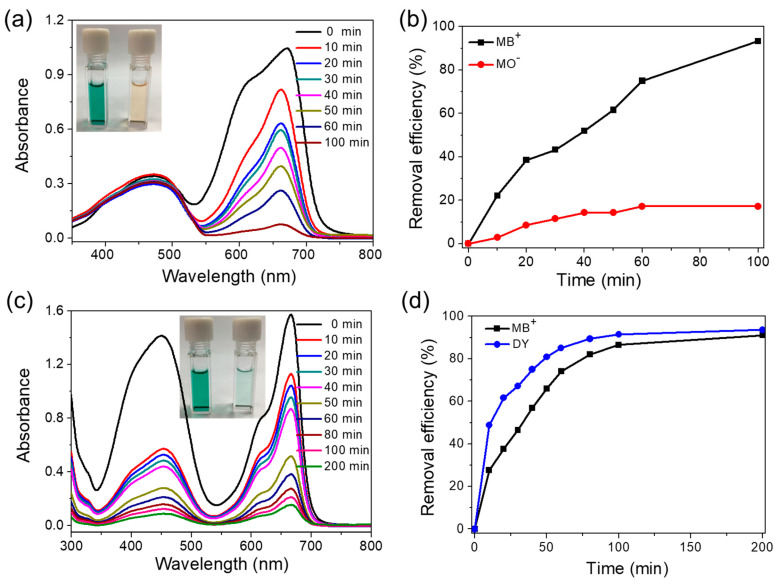
Time-dependent adsorption study of 20 mg/L^−1^ for each kind of cationic dye in 20 mL aqueous solution: (**a**) MB^+^/MO^−^ and (**b**) MB^+^/DY. The removal efficiency of each aqueous mixture: (**c**) MB^+^/MO^−^ and (**d**) MB^+^/DY. Inset photographs show the color change before and after dye adsorption (removal efficiency = (C_0_ − C_e_)/C_e_; C_0_: the original concentration; C_t_: the concentration at the moment t).

Meanwhile, it was found that the T-CMP could selectively adsorb mixtures of MB^+^/AO^−^ and MB^+^/DY by the meaningful structure; see Figure 5a–d. It was found that T-CMP only absorbs cationic-type dyes from the MB^+^/AO^−^ mixture solution; see Figure 5a. The solution color turned from dark green to light yellow, and the absorption peak at 655 nm dropped more violently; however, the peak at 460 nm remained unchanged, so the selective adsorption of anion and cationic dyes might have been achieved, as shown in Figure 5c. The mixture color of MB^+^/DY always changed from blue-green to colorless; therefore, the T-CMP could adsorb dyes from the solution for a long time, but has no selectivity for cationic or neutral dyes; see Figure 5b,d.

For further study and in order to establish the amount of the dye uptake capacities of T-CMP, the dye adsorption amounts are calculated using the equation R (mg g^−1^) = 20 mL × (C_0_ − C_t_)/3 mg, in which *R* is the dye uptake, C_0_ is the initial concentration (mg L^−1^), and C_e_ is the equilibrium concentration (mg L^−1^). As a result, the maximum uptake amounts of T-CMP were determined to be 132.47 mg g^−1^ for CV^+^, 128.32 mg g^−1^ for MB^+^, 123.29 mg g^−1^ for RB^+^, 123.60 mg g^−1^ for BR^+^, and 116.03 mg g^−1^ for DY, as shown in Figure S21. It was found that the T-CMP materials have large uptake capacities for cationic-type dyes by the rich N-donor and the microporous effect, or molecular interactions. This excellent performance might develop the T-CMP itself to be more advanced in the treatment of water pollution for target dyes.

**Figure 6:**
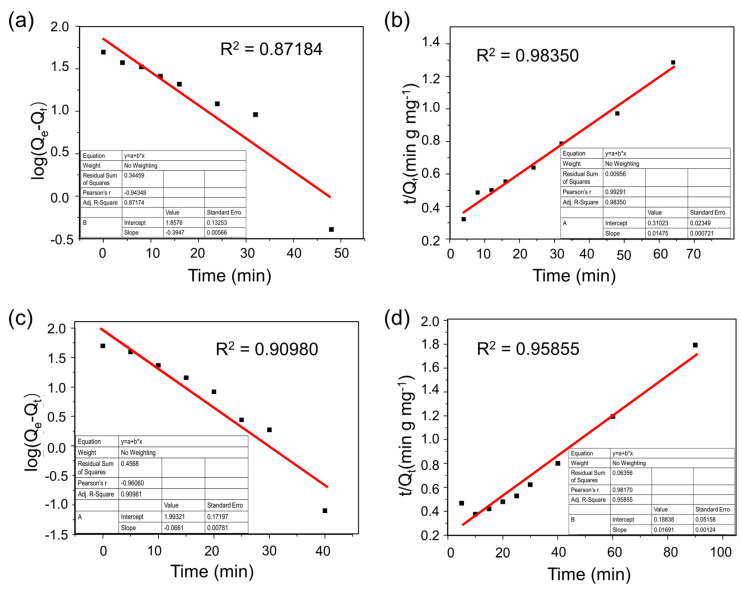
Pseudo-first-order kinetics (**a**) and pseudo-second-order kinetics (**b**) for adsorption of MB in the presence of T-CMP. Pseudo-first-order kinetics (**c**) and pseudo-second-order kinetics (**d**) for adsorption of MB in the presence of T-CMP-Me.

According to the results of the pseudo-first-order kinetic simulation and the pseudo-second-order kinetic simulation [41,42,43], shown in the Supplementary Information (Figure 6, Figure S22 and Table S3), it is found that the adsorption of MB by T-CMP and T-CMP-Me was more consistent with the quasi-second-order kinetic simulation. Considering that the adsorption rate is proportional to the number of unoccupied adsorption sites in the quasi-second-order kinetic model, the adsorption mechanism is assumed to be controlled by chemisorption. This result reveals that the porous materials T-CMP and T-CMP-Me adsorbed MB mainly by chemical adsorption. Considering that the T-CMP, T-CMP-Me, and organic dye molecules all have conjugate structures, we believe that the T-CMP and T-CMP-Me mainly adsorbed dye molecules by molecular interactions. By comparing the simulation results of the adsorption of MB by the T-CMP and T-CMP-Me with the pseudo-second-order kinetic constant *k_2_* and the equilibrium adsorption capacity *Q_e_*, it is found that T-CMP-Me has slightly faster adsorption but a smaller equilibrium adsorption capacity than T-CMP (Figure S23). The reason is that T-CMP-Me with positive charges is less stacked than T-CMP layers (Figure S4). The former increases the specific surface area and improves the adsorption rate, while the latter repulses dye molecules and reduces the adsorption sites.

**Figure 7:**
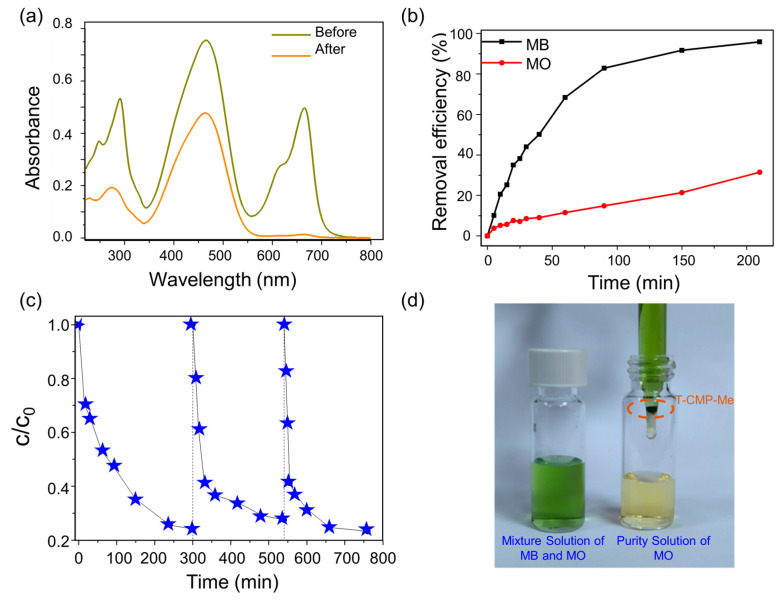
UV-vis absorption spectra (**a**) and adsorption rates (**b**) of the mixed aqueous solutions of MO and MB in the presence of T-CMP-Me. (**c**) The recycling performance of T-CMP-Me in the mixture. (**d**) The flow adsorption equipment and the photograph of flow adsorption equipment.

Furthermore, we carried out the adsorbent recycling experiment and found that both the polymer materials T-CMP and T-CMP-Me have good regeneration abilities (Figure 7). Subsequently, T-CMP-Me was selected as an adsorbent for adsorption in the mixed aqueous solution of MO and MB (Figure 7a), and it was found that it could selectively adsorb MB (Figure 7a,b). Finally, in order to simulate industrial sewage adsorption treatment, we built a set of simple flow adsorption devices (Figure 7c,d) to realize the selective adsorption of MB in a mixed aqueous solution of MO and MB, revealing that the material has good selective cyclic adsorption capacity.

## 5. Conclusions

In summary, triazine-conjugated microporous polymers with good dye adsorption properties were synthesized and characterized by the Sonogashira–Hagihara coupling reaction. The network polymers are found to have potential value for gas adsorption. All the materials underwent dye adsorption via the quasi-second-order kinetic simulation to give selective adsorption performance; more importantly, they displayed efficient removal abilities to adsorb cationic-type dyes and selectivity for the mixture solution. Finally, a set of simple flow adsorption devices was constructed, which will improve the triazine-conjugated microporous polymers (T-CMP and T-CMP-Me) as a new method for water pollution treatment.

## Data Availability

All data are included in the article.

## Supplementary Materials

The following are available online at https://www.mdpi.com/article/10.3390/molecules28124785/s1. Figure S1. ^1^H-NMR spectrum of TBPT. Figure S2. ^1^H-NMR spectrum of TEPT. Figure S3. Partial SEM images of T-CMP with different sizes. Figure S4. SEM and TEM images of T-CMP-Me. Figure S5. SEM images of T-CMP-Me with different sizes. Figure S6. Energy image of T-CMP. Figure S7. Powder X-ray diffraction spectrum of T-CMP and T-CMP-Me. Figure S8. The TG Curves of the molecules TBPT and TEPT, also compared with the porous materials T-CMP and T-CMP-Me. Figure S9. The pore size distribution obtained by the NLDFT method of T-CMP. Figure S10. The pore size distribution obtained by the NLDFT method of T-CMP-Me. Figure S11. H_2_ adsorption–desorption isotherms (77 K) of T-CMP. Figure S12. Time-dependent adsorption study of 20 mg/L^−1^ for each kind of MB^+^ dye in 20 mL aqueous solution for T-CMP-Me (3 mg). Figure S13. Time-dependent adsorption study of 20 mg/L^−1^ for each kind of CV^+^ dye in 20 mL aqueous solution for T-CMP-Me (3 mg). Figure S14. Time-dependent adsorption study of 20 mg/L^−1^ for each kind of RB^+^ dye in 20 mL aqueous solution for T-CMP-Me (3 mg). Figure S15. Time-dependent adsorption study of (20 mg/L^−1^ for each kind of MO dye in 20 mL aqueous solution for T-CMP-Me (3 mg). Figure S16. Time-dependent adsorption study of 20 mg/L^−1^ for each kind of AO dye in 20 mL aqueous solution for T-CMP-Me (3 mg). Figure S17. The removal efficiency of each aqueous mixture: (a) CV^+^, (b) MB^+^, (c) RB^+^, and (d) BR^+^ (removal efficiency = $\frac{C0-\mathrm{Ct}}{C0}\times 100\%$; C_0_: the original concentration; C_t_: the concentration at the moment t). Figure S18. Time-dependent adsorption study of 20 mg/L−1 for anionic dyes in 20 mL aqueous solution: (a) MO− and (b) AO−. The removal efficiency of anionic dyes: (c) MO− and (d) AO− (removal efficiency = (C0 − Ct)/C0 × 100%; C0: the original concentration; Ct: the concentration at the moment t). Figure S19. Time-dependent adsorption study of 20 mg/L−1 for neutral dye in 20 mL aqueous solution: (a) DY. The removal efficiency of anionic dyes: (b) DY (removal efficiency = (C0 − Ct)/C0×100%; C0: the original concentration; Ct: the concentration at the moment t). Figure S20. The contact angle images for T-CMP (left) and T-CMP-Me (right) with water. Figure S21. The maximum adsorption of dyes (CV^+^, MB^+^, RB^+^, BR^+^, and DY) of T-CMP. Figure S22. Time-dependent UV adsorption rates of MB+ dyes T-CMP and T-CMP-Me, respectively. Figure S23. Pseudo-second-order kinetics for adsorption of MO (a) and AO (b) in the presence of T-CMP-Me. Table S1. Data summary of energy spectrum analysis for T-CMP. Table S2. Data summary of the different kinds of dyes (label, structure, and charge). Table S3. Comparison of constants calculated based on respective pseudo-second-order kinetics.

## Sample Availability

Samples of Conjugated Microporous Polymers (CMPs) are available from the authors.

## References

[1] Lee J., Kim J.G., Chang J.Y. (2017). Fabrication of a conjugated microporous polymer membrane and its application for membrane catalysis. Sci. Rep..

[2] Luo S., Almatrafi E., Tang L., Song B., Zhou C., Zeng Y., Zeng G., Liu Z. (2022). Processable Conjugated Microporous Polymer Gels and Monoliths: Fundamentals and Versatile Applications. ACS Appl. Mater. Interfaces.

[3] Lee J.-S.M., Wu T.-H., Alston B.M., Briggs M.E., Hasell T., Hu C.-C., Cooper A.I. (2016). Porosity-engineered carbons for supercapacitive energy storage using conjugated microporous polymer precursors. J. Mater. Chem. A.

[4] Jiao Y., Wu F., Xie A., Wu L., Zhao W., Zhu X., Qi X. (2020). Electrically conductive conjugate microporous polymers (CMPs) via confined polymerization of pyrrole for electromagnetic wave absorption. Chem. Eng. J..

[5] Haleem A., Shafiq A., Chen S.-Q., Nazar M. (2023). A Comprehensive Review on Adsorption, Photocatalytic and Chemical Degradation of Dyes and Nitro-Compounds over Different Kinds of Porous and Composite Materials. Molecules.

[6] Li Q., Fan Z.-L., Xue D.-X., Zhang Y.-F., Zhang Z.-H., Wang Q., Sun H.-M., Gao Z., Bai J. (2018). A multi-dye@MOF composite boosts highly efficient photodegradation of an ultra-stubborn dye reactive blue 21 under visible-light irradiation. J. Mater. Chem. A.

[7] Amin K., Ashraf N., Mao L., Faul C.F.J., Wei Z. (2021). Conjugated microporous polymers for energy storage: Recent progress and challenges. Nano Energy.

[8] Holst J.R., Trewin A., Cooper A.I. (2010). Porous organic molecules. Nat. Chem..

[9] Jiang J.-X., Laybourn A., Clowes R., Khimyak Y.Z., Bacsa J., Higgins S.J., Adams D.J., Cooper A.I. (2010). High Surface Area Contorted Conjugated Microporous Polymers Based on Spiro-Bipropylenedioxythiophene. Macromolecules.

[10] Lee J.M., Cooper A.I. (2020). Advances in Conjugated Microporous Polymers. Chem. Rev..

[11] Chang Z., Zhang D.S., Chen Q., Bu X.H. (2013). Microporous organic polymers for gas storage and separation applications. Phys. Chem. Chem. Phys..

[12] Liu Z., Yin Y., Eginligil M., Wang L., Liu J., Huang W. (2021). Two-dimensional conjugated microporous polymer films: Fabrication strategies and potential applications. Polym. Chem..

[13] Chen L., Honsho Y., Seki S., Jiang D. (2010). Light-Harvesting Conjugated Microporous Polymers: Rapid and Highly Efficient Flow of Light Energy with a Porous Polyphenylene Framework as Antenna. J. Am. Chem. Soc..

[14] Sun Q., Aguila B., Song Y., Ma S. (2020). Tailored Porous Organic Polymers for Task-Specific Water Purification. Acc. Chem. Res..

[15] Tan Z., Su H., Guo Y., Liu H., Liao B., Amin A.M., Liu Q. (2020). Ferrocene-Based Conjugated Microporous Polymers Derived from Yamamoto Coupling for Gas Storage and Dye Removal. Polymers.

[16] Wang F., Sun W., Pan W., Xu N. (2015). Adsorption of sulfamethoxazole and 17β-estradiol by carbon nanotubes/CoFe_2_O_4_ composites. Chem. Eng. J..

[17] Li Y., Liu M., Chen L. (2017). Polyoxometalate built-in conjugated microporous polymers for visible-light heterogeneous photocatalysis. J. Mater. Chem. A.

[18] Tian S., Zhang J., Chen J., Kong L., Lu J., Ding F., Xiong Y. (2013). Fe_2_(MoO_4_)_3_ as an Effective Photo-Fenton-like Catalyst for the Degradation of Anionic and Cationic Dyes in a Wide pH Range. Ind. Eng. Chem. Res..

[19] Wang Z., Zhu C.-Y., Zhao H.-S., Yin S.-Y., Wang S.-J., Zhang J.-H., Jiang J.-J., Pan M., Su C.-Y. (2019). Record high cationic dye separation performance for water sanitation using a neutral coordination framework. J. Mater. Chem. A.

[20] Levard C., Hotze E.M., Lowry G.V., Brown G.E. (2012). Environmental Transformations of Silver Nanoparticles: Impact on Stability and Toxicity. Environ. Sci. Technol..

[21] Patel S., Hota G. (2016). Iron oxide nanoparticle-immobilized PAN nanofibers: Synthesis and adsorption studies. RSC Adv..

[22] Jiang J.X., Su F., Trewin A., Wood C.D., Campbell N.L., Niu H., Dickinson C., Ganin A.Y., Rosseinsky M.J., Khimyak Y.Z. (2007). Conjugated microporous poly(aryleneethynylene) networks. Angew. Chem. Int. Ed..

[23] Cheng G., Hasell T., Trewin A., Adams D.J., Cooper A.I. (2012). Soluble conjugated microporous polymers. Angew. Chem. Int. Ed..

[24] Luo L., Wu Z., Wu Z., Liu Y., Huang X., Ling R., Ye L., Luo X., Wang C. (2022). Role of Structure in the Ammonia Uptake of Porous Polyionic Liquids. ACS Sustain. Chem. Eng..

[25] Haleem A., Pan J.-M., Shah A., Hussain H., He W. (2023). A systematic review on new advancement and assessment of emerging polymeric cryogels for environmental sustainability and energy production. Sep. Purif. Technol..

[26] Hu G., Wu T., Liu Z., Gao S., Hao J. (2023). Application of Molecular Imprinting Technology Based on New Nanomaterials in Adsorption and Detection of Fluoroquinolones. Anal. Methods.

[27] Mohanan M., Ahmad H., Ajayan P., Pandey P.K., Calvert B.M., Zhang X., Chen F., Kim S.J., Kundu S., Gavvalapalli N. (2023). Using molecular straps to engineer conjugated porous polymer growth, chemical doping, and conductivity. Chem. Sci..

[28] Segura J.L., Mancheno M.J., Zamora F. (2016). Covalent organic frameworks based on Schiff-base chemistry: Synthesis, properties and potential applications. Chem. Soc. Rev..

[29] Schwarz D., Acharjya A., Ichangi A., Kochergin Y.S., Lyu P., Opanasenko M.V., Tarabek J., Vacek Chocholousova J., Vacek J., Schmidt J. (2019). Tuning the Porosity and Photocatalytic Performance of Triazine-Based Graphdiyne Polymers through Polymorphism. ChemSusChem.

[30] Luo D., Li M., Ma Q., Wen G., Dou H., Ren B., Liu Y., Wang X., Shui L., Chen Z. (2022). Porous organic polymers for Li-chemistry-based batteries: Functionalities and characterization studies. Chem. Soc. Rev..

[31] Zhou Z., Shinde D.B., Guo D., Cao L., Nuaimi R.A., Zhang Y., Enakonda L.R., Lai Z. (2021). Flexible Ionic Conjugated Microporous Polymer Membranes for Fast and Selective Ion Transport. Adv. Funct. Mater..

[32] Zou Y., Wang D., Guo J., Yang J., Pu Y., Chen J.-F. (2022). Synthesis of poly(9,9-dioctylfluorene) in a rotating packed bed with enhanced performance for polymer light-emitting diodes. Polym. Chem..

[33] Liu Y., Cui Y., Zhang C., Du J., Wang S., Bai Y., Liang Z., Song X. (2018). Post-cationic Modification of a Pyrimidine-Based Conjugated Microporous Polymer for Enhancing the Removal Performance of Anionic Dyes in Water. Chem. Eur. J..

[34] Yuan Y., Huang H., Chen L., Chen Y. (2017). N,N′-Bicarbazole: A Versatile Building Block toward the Construction of Conjugated Porous Polymers for CO_2_ Capture and Dyes Adsorption. Macromolecules.

[35] Tantisriyanurak S., Duguid H.N., Peattie L., Dawson R. (2020). Acid Functionalized Conjugated Microporous Polymers as a Reusable Catalyst for Biodiesel Production. ACS Appl. Poly. Mater..

[36] Zou L.H., Johansson A.J., Zuidema E., Bolm C. (2013). Mechanistic insights into copper-catalyzed Sonogashira-Hagihara-type cross-coupling reactions: Sub-mol% catalyst loadings and ligand effects. Chem. Eur. J..

[37] Wang Y., Zhong H., Li L., Wang R. (2016). Facile Synthesis and Tunable Porosities of Imidazolium-Based Ionic Polymers that Contain In Situ Formed Palladium Nanoparticles. ChemCatChem.

[38] Soliman A.B., Haikal R.R., Hassan Y.S., Alkordi M.H. (2016). The potential of a graphene-supported porous-organic polymer (POP) for CO_2_ electrocatalytic reduction. Chem. Commun..

[39] Alkordi M.H., Haikal R.R., Hassan Y.S., Emwas A.-H., Belmabkhout Y. (2015). Poly-functional porous-organic polymers to access functionality—CO_2_ sorption energetic relationships. J. Mater. Chem. A.

[40] Li Q., Xue D.-X., Zhang Y.-F., Zhang Z.-H., Gao Z., Bai J. (2017). A dual-functional indium–organic framework towards organic pollutant decontamination via physically selective adsorption and chemical photodegradation. J. Mater. Chem. A.

[41] Liu Y. (2009). Is the Free Energy Change of Adsorption Correctly Calculated?. J. Chem. Eng. Data.

[42] Ho Y.S., McKay G. (1999). Pseudo-second order model for sorption processes. Process Biochem..

[43] Wang S., Meng X., Luo H., Yao L., Song X., Liang Z. (2022). Post-synthetic modification of conjugated microporous polymer with imidazolium for highly efficient anionic dyes removal from water. Sep. Purif. Technol..

