# Supplementary material for: Electron-Rich Triazine-Conjugated Microporous Polymers for the Removal of Dyes from Wastewater
Source: Molecules. 2023 Jun 15;28(12):4785. doi: 10.3390/molecules28124785 (PMC10301975; doi:10.3390/molecules28124785)
Supplement: Supplementary file 1 [file molecules-28-04785-s001.zip › molecules-2430687-supplementary.pdf]

Electron-Rich Triazine-Conjugated Microporous Polymers for the Removal of Dyes from Wastewater

Bao-Ning Li [1,*,†], Xing-Long Zhang [1,†], Xiao-Hui Bai [1,†], Zhen-Jie Liang [2], Jian Li [1] and Xiao-Yong Fan [1]

[1] School of Chemistry and Chemical Engineering, Yulin University, Yulin 719000, China;
lbningyl@163.com (X.-L.Z.); bxh0727@163.com (X.-H.B.); lijian5220@163.com (J.L.);
fanxy@yulinu.edu.cn (X.-Y.F.)

[2] School of Chemistry, Sun Yat-Sen University, Guangzhou 510006, China; liangzhj9@mail2.sysu.edu.cn

* Correspondence: lbning@yulinu.edu.cn

† These authors contributed equally.

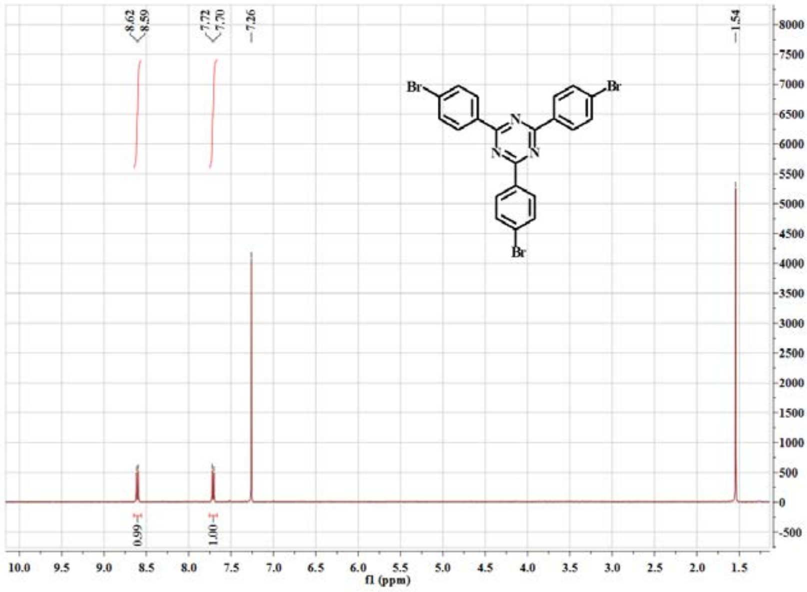

Figure S1. ^{1}H-NMR spectrum of TBPT, (400 MHz, $CDCl_3$, TMS): δ (ppm) 8.58 (d, 6H), 7.69 (d, 6H).

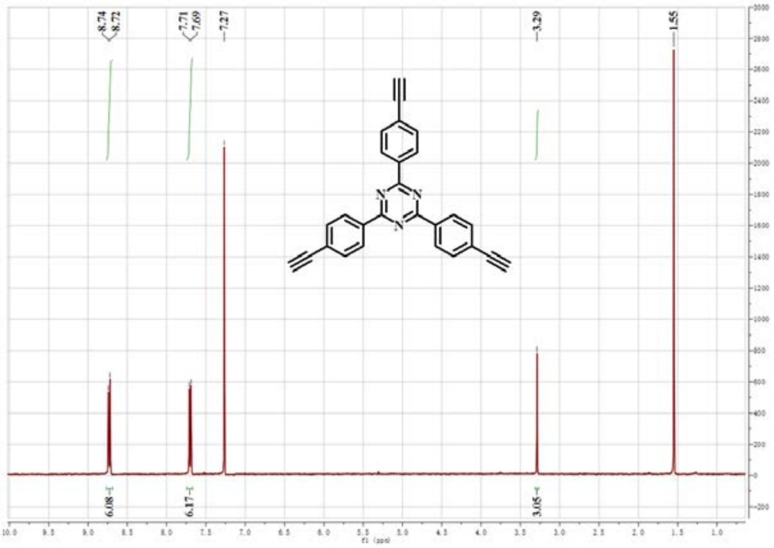

Figure S2. ^{1}H-NMR spectrum of TEPT, (400 MHz, $CDCl_3$, TMS): δ (ppm) 8.73 (d, 6H), 7.70 (d, 6H), 3.29 (s, 3H).

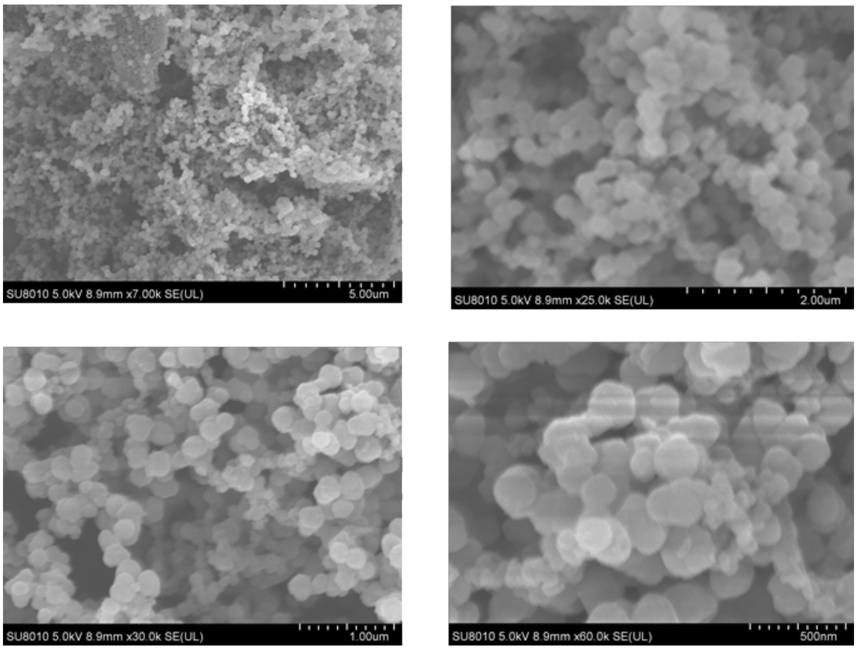

Figure S3. SEM-images of T-CMP with different size.

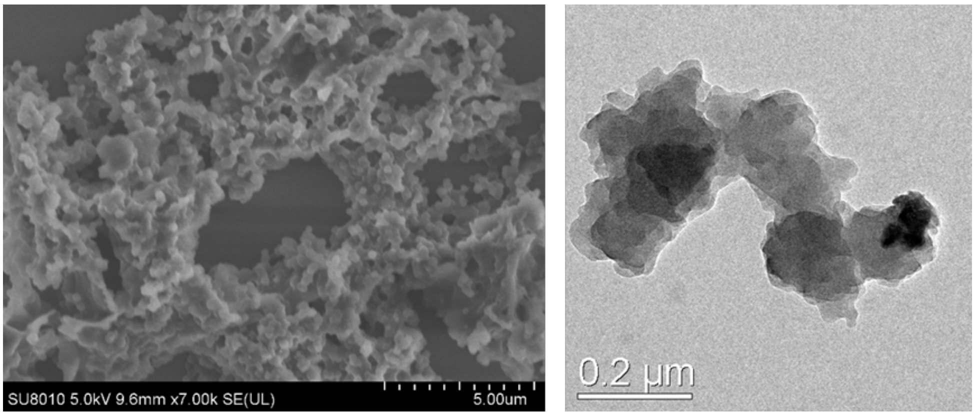

Figure S4. SEM and TEM images of T-CMP-Me.

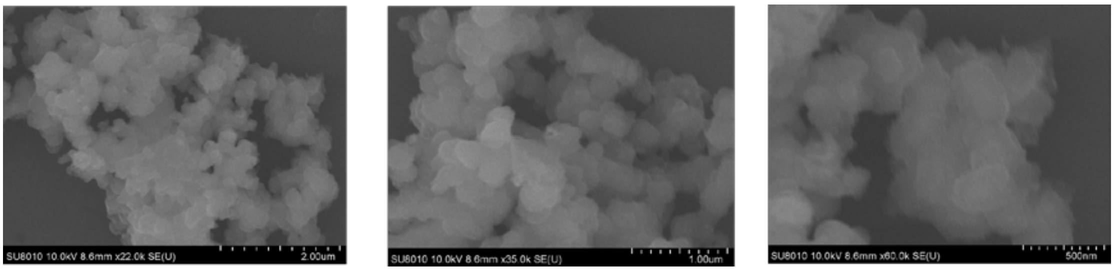

Figure S5. SEM images of T-CMP-Me with different size.

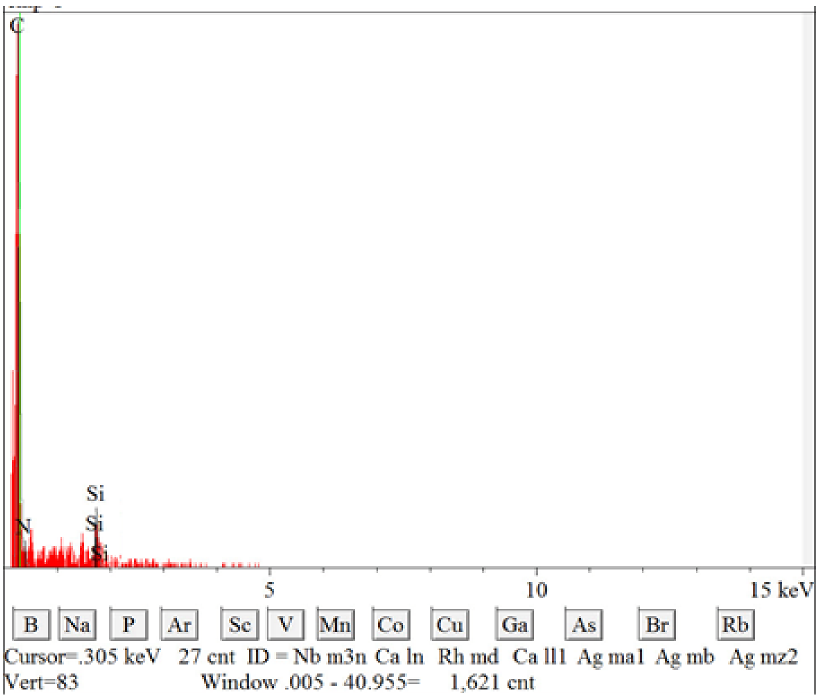

Figure S6. Energy-image of T-CMP.

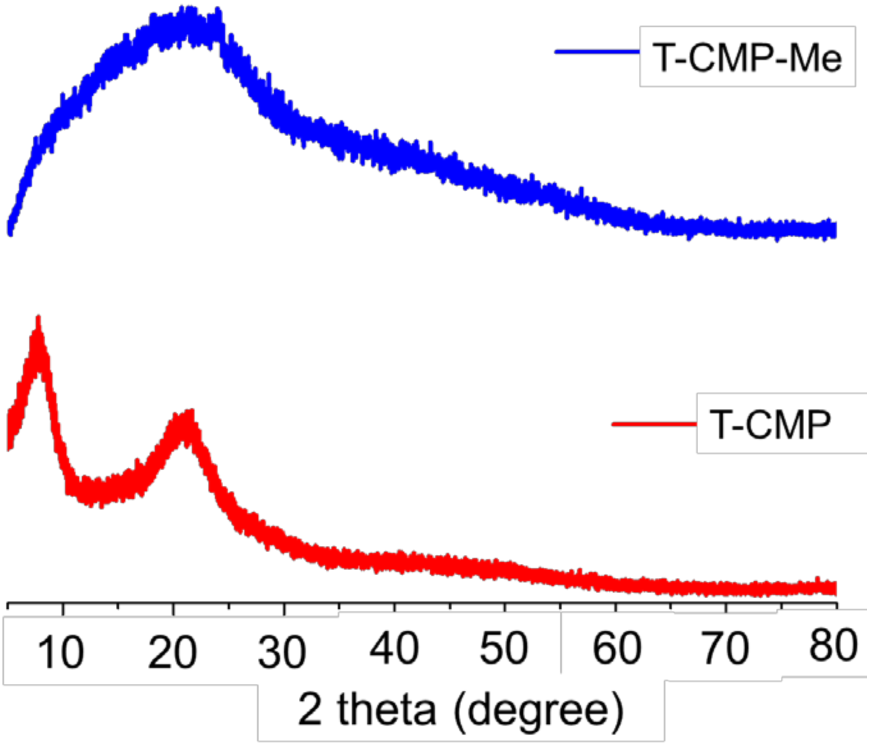

Figure S7. Powder x-ray diffraction spectrum of T-CMP and T-CMP-Me.

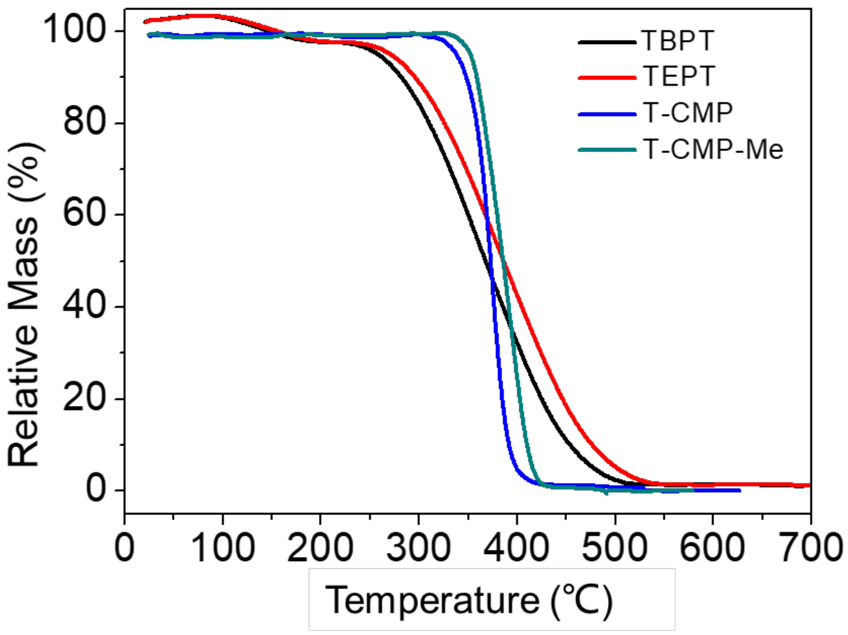

Figure S8. The TG-Curves of the molecules TBPT and TEPT, also compared with the porous materials T-CMP and T-CMP-Me.

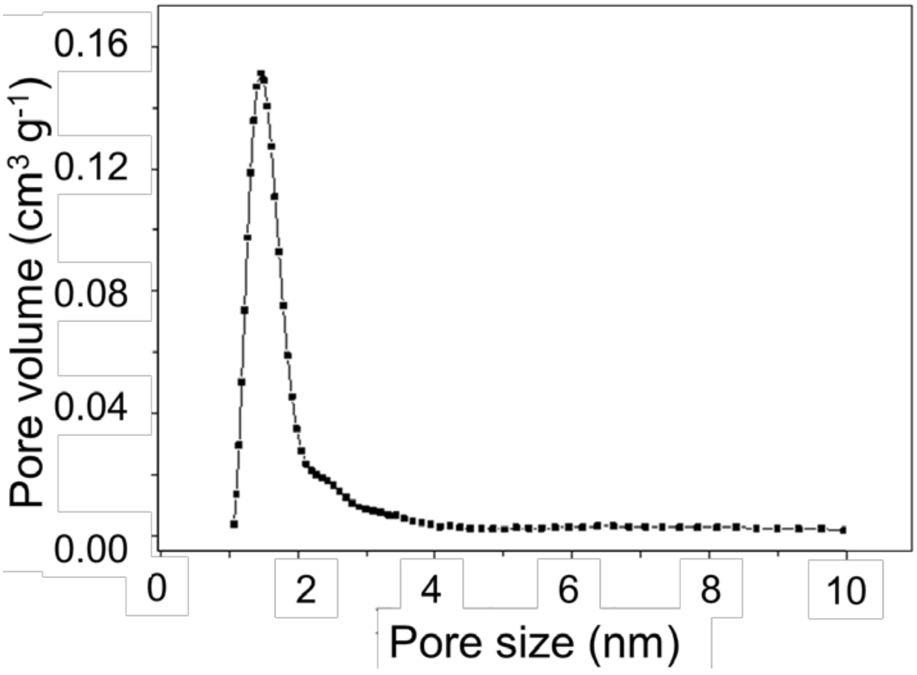

Figure S9. The pore size distribution obtained by the NLDFT method of T-CMP.

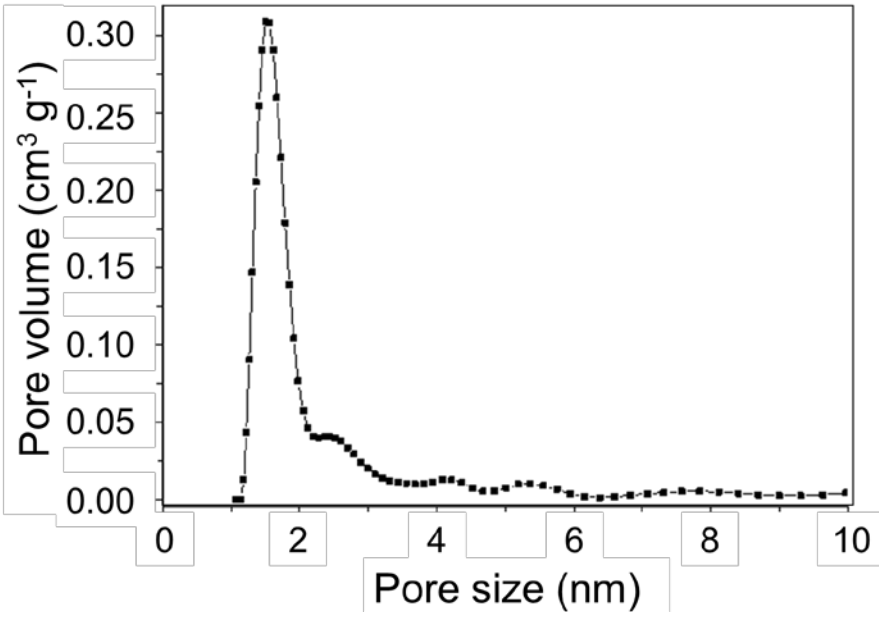

Figure S10. The pore size distribution obtained by the NLDFT method of T-CMP-Me.

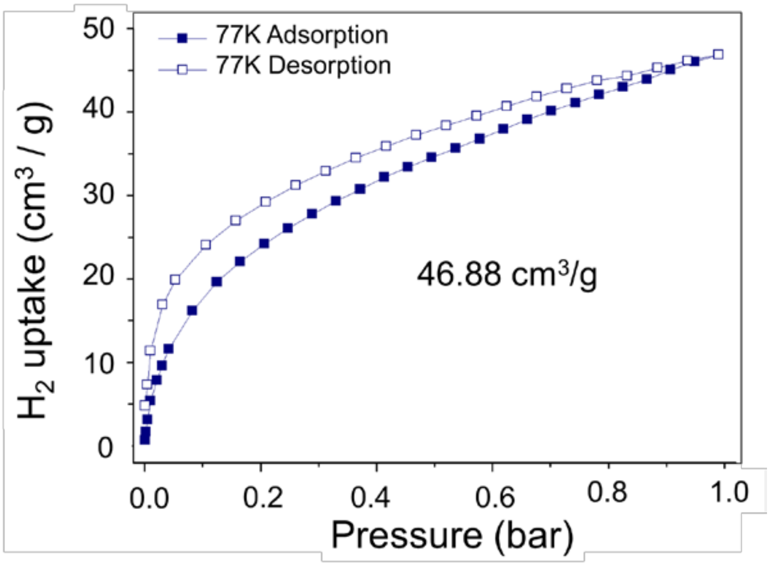

Figure S11. H_2 adsorption–desorption isotherms (77 K) of T-CMP.

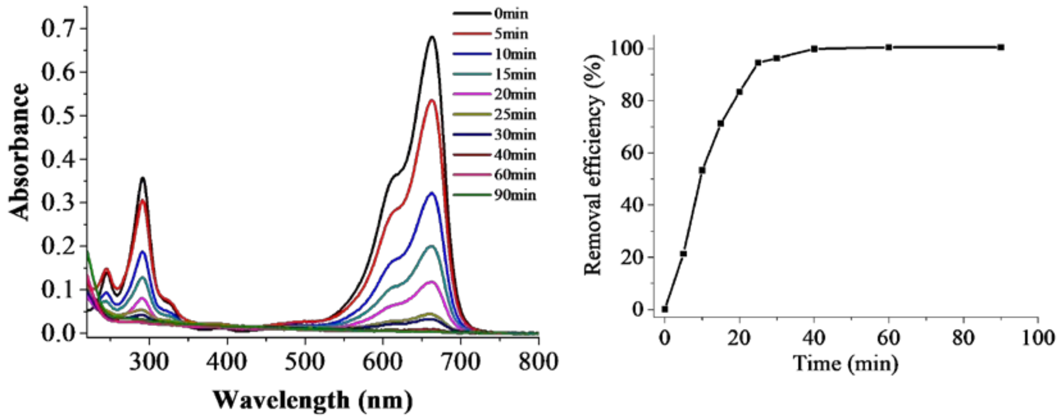

Figure S12. Time-dependent adsorption study of (20 mg/L^{-1} for each kind of MB^+ dyes, in 20 mL) aqueous solution for T-CMP-Me (3 mg).

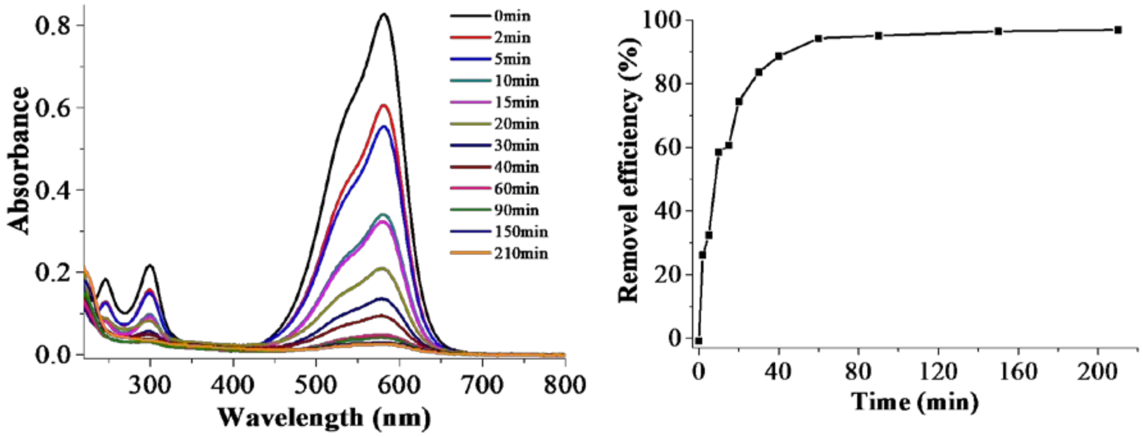

Figure S13. Time-dependent adsorption study of (20 mg/L^{-1} for each kind of CV^+ dyes, in 20 mL) aqueous solution for T-CMP-Me (3 mg).

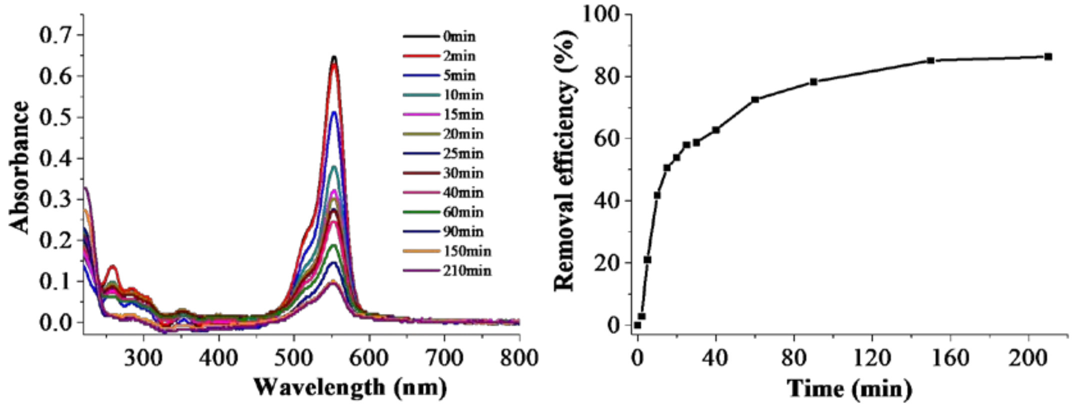

Figure S14. Time-dependent adsorption study of (20 mg/L^{-1} for each kind of RB^+ dyes, in 20 mL) aqueous solution for T-CMP-Me (3 mg).

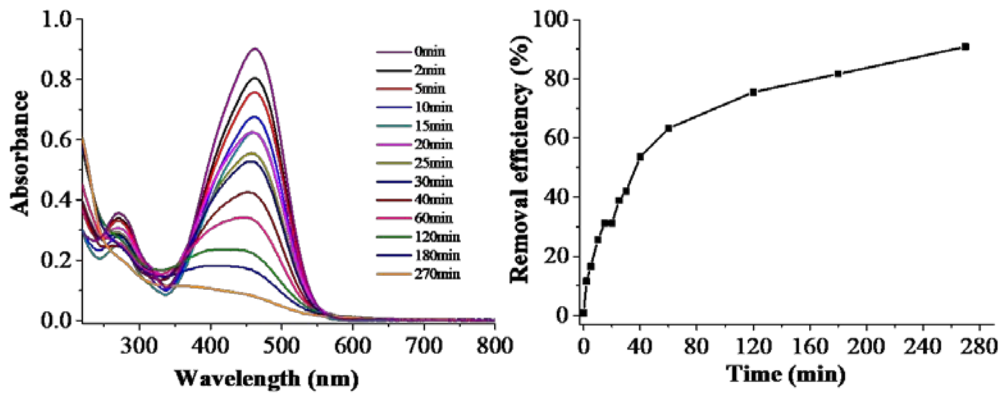

Figure S15. Time-dependent adsorption study of (20 mg/L^{-1} for each kind of MO dyes, in 20 mL) aqueous solution for T-CMP-Me (3 mg)

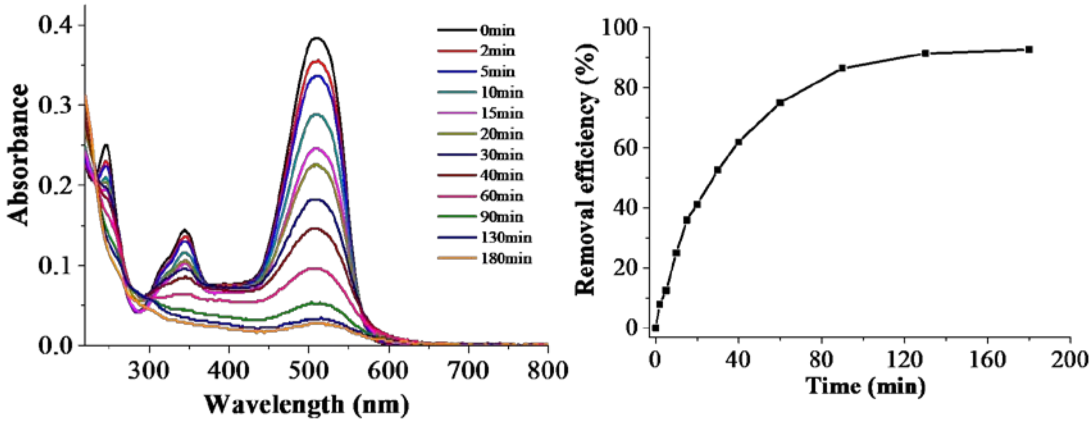

Figure S16. Time-dependent adsorption study of (20 mg/L^{-1} for each kind of AO dyes, in 20 mL) aqueous solution for T-CMP-Me (3 mg).

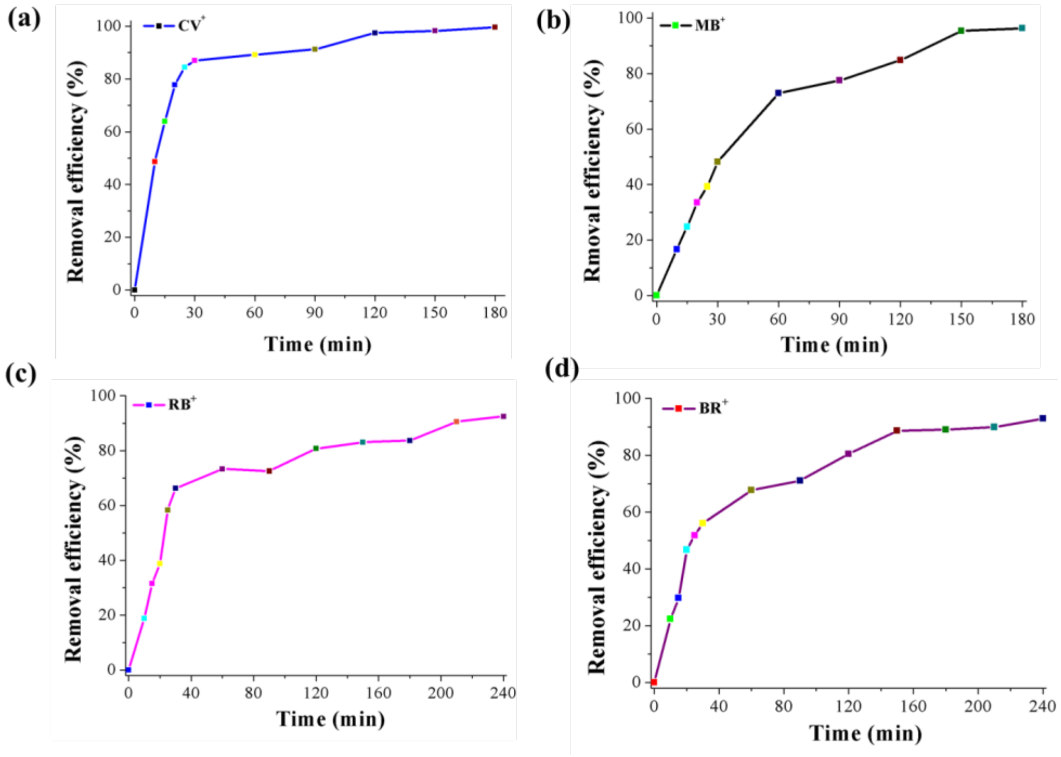

Figure S17. The removal efficiency of each mixture aqueous: (a) CV^+, (b) MB^+, (c) RB^+, (d) BR^+. (Removal efficiency = $\frac{Co-Ct}{Co} \times 100\%$, C_0: the original concentration; C_t: the concentration at the moment t).

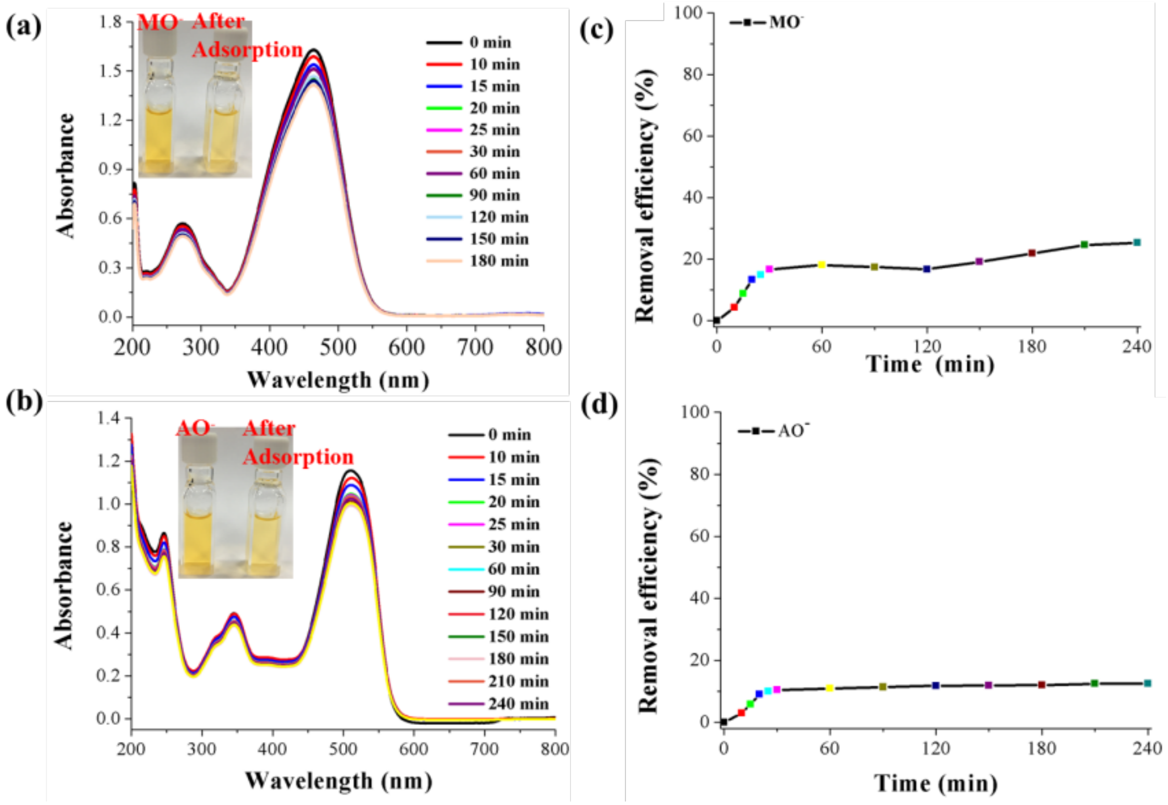

Figure S18. Time-dependent adsorption study of (20 mg/L^{-1} for anionic-dyes, in 20 mL) aqueous solution: (a) MO^-, (b) AO^-. The removal efficiency of anionic dyes: (c) MO^-, (d) AO^-. (Removal efficiency = $(C_0-C_t)/C_0 \times 100\%$, C_0: the original concentration; C_t: the concentration at the moment *t*).

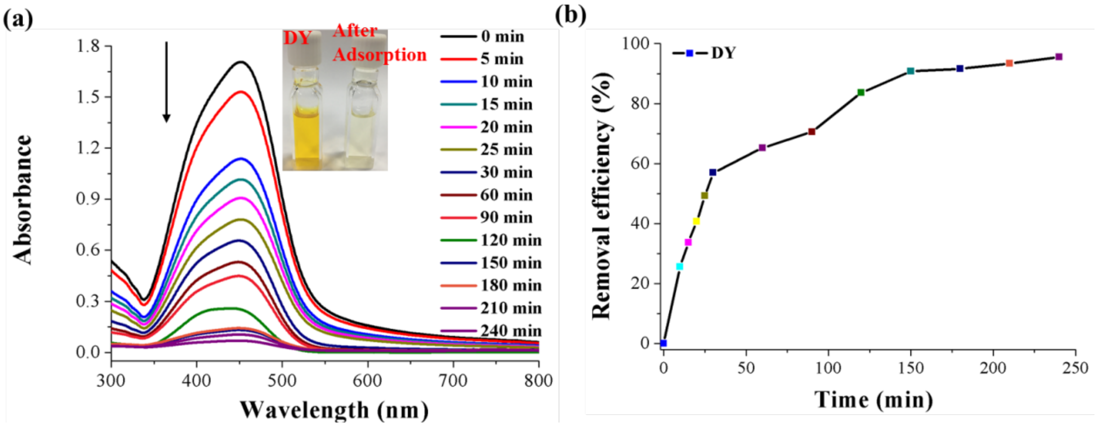

Figure S19. Time-dependent adsorption study of (20 mg/L^{-1} for neutral-dye, in 20 mL) aqueous solution: (a) DY. The removal efficiency of anionic dyes: (b) DY. (Removal efficiency = $(C_0-C_t)/C_0 \times 100\%$, C_0: the original concentration; C_t: the concentration at the moment *t*).

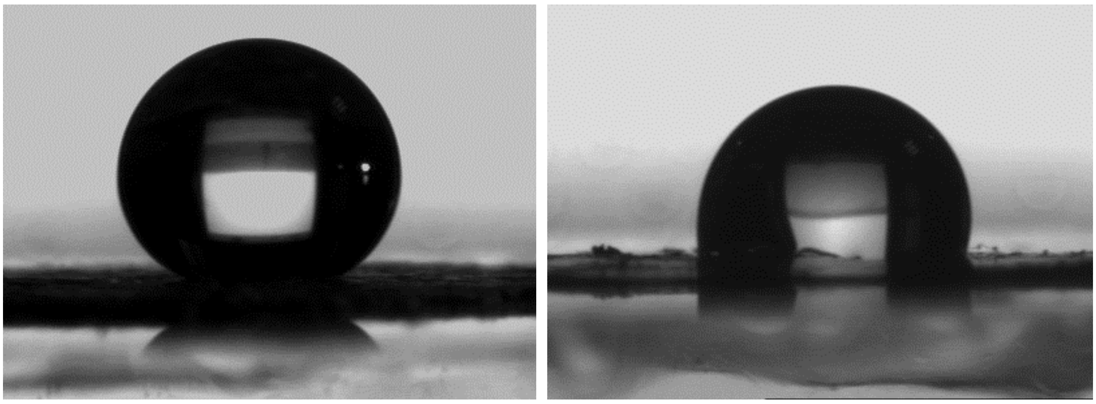

Figure S20. The Contact angle images for T-CMP (left) and T-CMP-Me (right) with water.

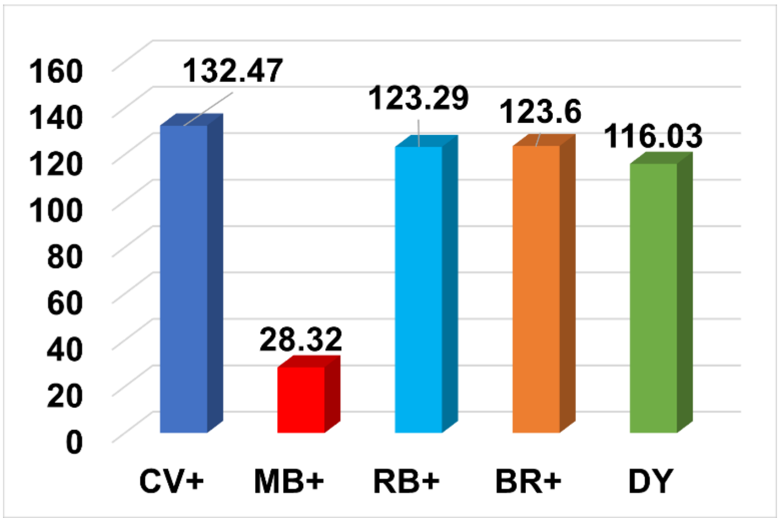

Figure S21. The maximum adsorption of dyes (CV^+, MB^+, RB^+, BR^+, DY) of T-CMP.

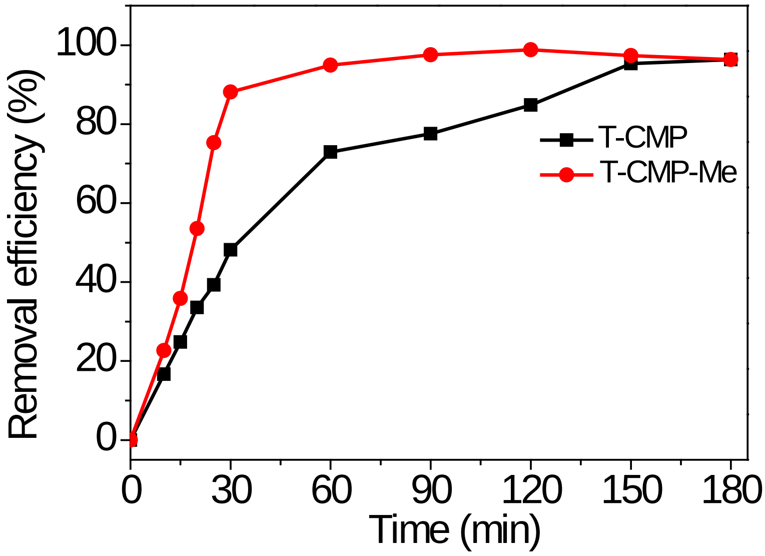

Figure S22. Time-dependent UV adsorption rates of MB^+ dyes T-CMP and T-CMP-Me, respectively.

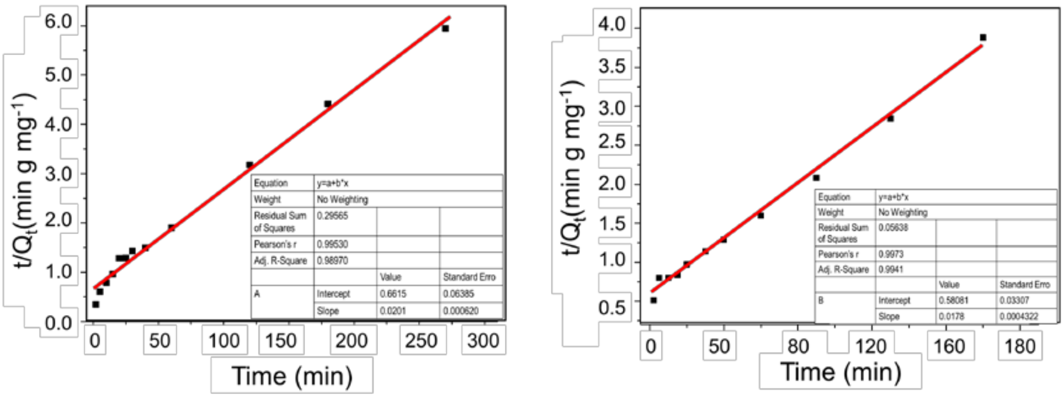

(a) **(b)**

Figure S23. Pseudo-second-order kinetics for adsorption of MO (a) and AO (b) in the presence of T-CMP-Me.

Table S1. Data summary energy spectrum analysis for T-CMP.

Elt.	Line	Intensity (c/s)	Atomic %	Conc	Units	
C	Ka	1.86	14.229	6.780	wt.%	
N	Ka	.51	4.210	2.340	wt.%	
Si	Ka	29.67	81.561	90.880	wt.%	
			100.000	100.000	wt.%	Total

Table S2. Data summary of the different kinds of dyes (label, structure, charge).

	Neutral	Anionic		Cationic			
**Name**	**DY**	**AO$^-$**	**MO$^-$**	**MB$^+$**	**CV$^+$**	**BR$^+$**	**RB$^+$**
**Structure**	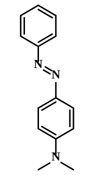	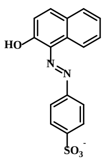	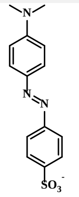	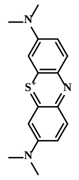	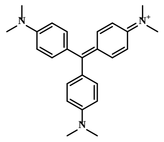	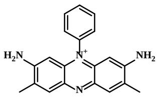	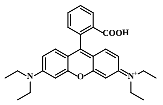
**Charge**	**0**	**-1**	**-1**	**+1**	**+1**	**+1**	**+1**

DY: dimethyl yellow; AO: Acid orange; MO: methyl orange; MB: methylene blue; CV: crystal violet; BR: basic red; RB: rhodamine B.

Table S3. Comparison of constans calculated based on respective pseudo-second-order kinetics.

	T-CMP		T-CMP-Me	
	Q_e	K_2	Q_e	K_2
MB	67.80	0.000701	59.14	0.00152
CV	51.79	0.00232	50.51	0.00279
RB	51.02	0.000651	46.51	0.00139
MO	-*	-*	49.75	0.000611
AO	-*	-*	56.18	0.000546

The symbol * stands for meaningless and cannot be fitted correctly.
